# The Sleep and Recovery Practices of Athletes

**DOI:** 10.3390/nu13041330

**Published:** 2021-04-17

**Authors:** Rónán Doherty, Sharon M. Madigan, Alan Nevill, Giles Warrington, Jason G. Ellis

**Affiliations:** 1 Sports Lab North West, Letterkenny Institute of Technology, Port Road, Letterkenny, F92 FC93 Donegal, Ireland; 2 Sport Ireland Institute, National Sport Campus, Abbotstown, D15 PNON Dublin, Ireland; smadigan@instituteofsport.ie; 3 Northumbria Centre for Sleep Research, Northumbria University, Newcastle NE7 7XA, UK; jason.ellis@northumbria.ac.uk; 4 Faculty of Education, Health and Wellbeing, University of Wolverhampton, Walsall Campus, Walsall WV1 1LY, UK; a.m.nevill@wlv.ac.uk; 5 Health Research Institute, Schuman Building, University of Limerick, V94 T9PX Limerick, Ireland; Giles.Warrington@ul.ie; 6 Department of Physical Education and Sport Sciences, University of Limerick, V94 T9PX Limerick, Ireland

**Keywords:** sleep, recovery, nutrition, alcohol, athletes

## Abstract

Background: Athletes maintain a balance between stress and recovery and adopt recovery modalities that manage fatigue and enhance recovery and performance. Optimal TST is subject to individual variance. However, 7–9 h sleep is recommended for adults, while elite athletes may require more quality sleep than non-athletes. Methods: A total of 338 (elite *n* = 115, 74 males and 41 females, aged 23.44 ± 4.91 years; and sub-elite *n* = 223, 129 males and 94 females aged 25.71 ± 6.27) athletes were recruited from a variety of team and individual sports to complete a battery of previously validated and reliable widely used questionnaires assessing sleep, recovery and nutritional practices. Results: Poor sleep was reported by both the elite and sub-elite athlete groups (i.e., global PSQI score ≥5—elite 64% [*n* = 74]; sub-elite 65% [*n* = 146]) and there was a significant difference in sport-specific recovery practices (3.22 ± 0.90 vs. 2.91 ± 0.90; *p* < 0.001). Relatively high levels of fatigue (2.52 ± 1.32), stress (1.7 ± 1.31) and pain (50%, *n* = 169) were reported in both groups. A range of supplements were used regularly by athletes in both groups; indeed, whey (elite *n* = 22 and sub-elite *n* = 48) was the most commonly used recovery supplement in both groups. Higher alcohol consumption was observed in the sub-elite athletes (12%, *n* = 26) and they tended to consume more units of alcohol per drinking bout. Conclusion: There is a need for athletes to receive individualised support and education regarding their sleep and recovery practices.

## 1. Introduction

Post-exercise recovery is vital for all athletes and the balance between training stress and physical recovery must be managed to maximise the adaptation from, and performance in, subsequent training sessions or competitions [1,2]. The repetitive demanding nature of a competitive season can test athletes’ physiological and psychological capacity. Athletes must maintain a balance between stress and recovery and adopt recovery modalities that manage fatigue and enhance recovery and performance in subsequent training/competition [1]. The regulation of performance during exercise has increasingly been interpreted as a cohesive, multifaceted process involving both the central nervous system (CNS) and the peripheral nervous system (PNS) [3,4]. While there is debate on whether the regulation of exercise performance is derived primarily from the CNS or PNS [5] and whether the regulation is conscious [6] or anticipatory [7], changing CNS drive and motor unit recruitment is widely considered to be associated with fatigue (i.e., reduced physical and mental capacity) [3]. In contrast, physical fatigue has many potential drivers (dehydration, glycogen depletion, muscle damage and mental fatigue), and recovery of muscle function is predominantly a matter of reversing the main causes of fatigue. Sleep deprivation (<7 h) increases circulating stress hormones (e.g., cortisol) [8]; decreases the regeneration of carbohydrate stores (i.e., glycogen) [9]; deregulates appetite and impacts on energy expenditure [10]; increases catabolism and reduces anabolism, impacting the rate of muscle repair (MPS) [11,12]. Therefore, sleep plays a key role in facilitation of post-exercise recovery or the reduction in fatigue and the reversal of the processes that lead to fatigue [13].

Athletes experience stress for various reasons (e.g., training, competition, travel and lifestyle) including periods of both acute and residual fatigue due to heavy training and competition schedules [14]. For example, field-based team sports are characterised by repeated bouts of intermittent activity (sprinting) with short rest periods, representing high physiological stress [15], neuromuscular stress [16,17] and high rates of perceived exertion (i.e., how hard exercise seems) [18]. Further, individual endurance athletes experience fatigue due to prolonged activity, resulting in glycogen depletion, thermal stress and/or dehydration [19]. Relative stress is accumulated when successive bouts of training are combined with suboptimal recovery (under-recovery) impacting subsequent performance in training and competition [20]. It has been suggested that decreasing the natural timeframe of the bodies’ regenerative processes via recovery strategies is vital for performance [21]. Such recovery strategies can be divided into physiological strategies (e.g., sleep, cold water immersion, cryotherapy, contrast therapy, massage and compression), pharmacological (e.g., non-steroidal anti-inflammatory drugs [NSAIDs]) and nutritional (e.g., nutrient timing, composition and supplementation) [22]. However, it must be noted that some research has suggested that interfering with the body’s natural recovery processes, particularly inflammatory responses and OS, could reduce training adaptations [23]. A recent review addressed these concerns in relation to the application of nutritional strategies to reduce muscle damage [24].

Sleep has previously been self-reported as the most important recovery modality utilised by both elite and sub-elite athletes [1,25,26]. Furthermore, it has been suggested that sleep was a new frontier in performance enhancement for athletes [27]. Sleep has a restorative effect on the immune system and the endocrine system [28,29,30], facilitates the recovery of the nervous and metabolic cost of the waking state and has an integral role in cognitive function [31]. The relationship between sleep, nutrition and recovery is an emerging area of interest [26,32,33,34,35,36,37,38,39,40,41,42,43,44,45]. Sleep has two basic states—non-rapid eye movement sleep (NREM) and rapid eye movement (REM) sleep. NREM is subdivided into three stages based on a continuum from light sleep (Stage N1 and N2) to deep sleep (Stage N3). It has been hypothesised that sleep, especially slow-wave sleep (Stage N3), is vital for physical recovery, due to the relationship with growth hormone release [44,46]. The National Sleep Foundation has proposed 12 indicators of sleep quality including 4 sleep continuity variables (sleep latency, awakenings >5 min, wake after sleep onset and sleep efficiency), 5 sleep architecture variables (REM sleep, N1 sleep, N2 sleep, N3 sleep and arousals) and 3 nap-related variables (naps per 24 h, nap duration and days per week with at least one nap) [47]. Sleep can be considered adequate when there is no daytime sleepiness or dysfunction.

For sleep to have a restorative effect on the body, it must be of adequate duration, quality, and appropriately timed [38,48]. The National Sleep Foundation has produced guidelines regarding sleep duration for adolescents (recommended 8–10 h), adults (recommended 7–9 h), and older adults (7–8 h) [48]. It has been argued that elite athletes may require more quality sleep than non-athletes [49]. It has recently been suggested that a one-size-fits-all sleep recommendation (7–9 h) may be inappropriate for athlete performance and health and an individual approach should be adapted including an assessment of perceived sleep needs [50].

Sleep inadequacy is common in athletes and can be attributed to the lack of an appropriate sleep routine due to changing training schedules, timetables and other sleep-incompatible behaviours, e.g., late night blue-light exposure [26,50]. Previous research has reported sleep durations <7 h [51], long sleep onset latency [26,52], daytime sleepiness [53], and daytime fatigue [54]. Studies investigating sleep quality in elite athletes have demonstrated that 50–80% experience sleep disturbance and 22–26% experience highly disturbed sleep [37,53,55]. Irregular sleep–wake patterns influence the homeostatic and circadian regulation of sleep, which reduces both sleep quality and quantity [56]. For athletes, post-completion routines and heightened arousal (i.e., medical care, recovery strategies, meals, media commitments and travel) can lead to later bedtimes, which can adversely affect sleep quality and quantity. Reduced sleep is associated with increased catabolic and reduced anabolic hormones, which results in impaired muscle protein synthesis [12], potentially blunting training adaptations and recovery.

Sleep disorders are identified by a wide range of symptoms that impact health and quality of life [57], cognitive performance [58] and physical performance [25,59]. Over 80 sleep disorders are listed in the third edition of the International Classification of Sleep Disorders (ISCD-3) [60]. The ICSD-3 includes seven major categories of sleep disorders: insomnia, sleep-related breathing disorders, central disorders of hypersomnolence, circadian rhythm sleep wake disorders (CRSWDs), sleep-related movement disorders, parasomnias and other sleep disorders [60]. In the general population, the most common sleep disorders are obstructive sleep apnoea (OSA), insomnia and restless legs syndrome (RLS) [61]. Sleep-related breathing disorders are characterised by breathing issues during sleep [62]. OSA is a frequent condition characterised by repeated episodes of partial or complete reduction in breathing activity during sleep [63]. Insomnia is characterised by difficulty falling asleep, staying asleep, waking too early with daytime symptoms of fatigue, resistance to going to bed and/or difficulty sleeping without intervention occurring at least 3 times per week over a period of one month ([64,65]. Central disorders of hypersomnolence are typified by excessive daytime sleepiness that cannot be attributed to another sleep disorder [60]. CRSWDs are chronic (≥3 months) patterns of sleep–wake disruption caused by an alteration to the endogenous circadian or desychronisation of the circadian rhythm and the sleep–wake schedule, causing sleep–wake disturbance and distress or impairment [60]. Sleep-related movement disorders may result from an unpleasant crawling, deep-aching sensation in the legs or arms that is relieved through movement [66]. Parasomnias are undesirable movements or behaviours that occur during sleep, e.g., sleep walking, sleep talking, night terrors and REM sleep behaviour disorder [66]. Other sleep disorders include all sleep disorders that do not meet the criteria for another sleep disorder classifications [62].

Polysomnography (PSG) is the ‘gold-standard’ method of sleep assessment and records sleep continuity, sleep architecture and REM sleep. A common global approach to the assessment of sleep quality is the use of self-report ratings reflecting an individual’s satisfaction with their sleep [47,67]. Sleep continuity is commonly assessed using sleep diaries and measures include time the subject went to bed, time the subject tried to initiate sleep, the length of time from turning off the lights until sleep onset (sleep onset latency), number and duration of awakenings, the degree of sleep maintenance during the night (sleep efficiency or the ratio of wake time to time in bed; awake time after sleep onset) sleep duration (total sleep time), time the subject woke up, time the subject got out of bed and sleep quality (subjective rating of sleep) [68,69].

Actigraphy is also used to assess sleep, regularly in combination with sleep diaries. Actigraphy involves wearing a small monitor (usually on the non-dominant wrist) which records body movement, high levels of activity are used as a measure of wakefulness and low levels of activity are classified as sleep [69]. Activity monitors record movement as a function of time [70], typically a tri-axial accelerometer is used to determine sleep/wake based on a proprietary algorithm [71]. A limitation of actigraphy is that all activity is recorded as waking unless the sleep diaries show an attempt to sleep (i.e., lying down trying to sleep) and the activity counts are low enough to indicate the subject is stationary [32]. However, actigraphy has been shown to be reliable and valid in relation to PSG for general measures of sleep [72,73].

Athletes’ schedules can negatively impact their sleep and recovery [51,52], and the repetitive demanding nature of a competitive season can also test athletes’ physiological and psychological capacity, reinforcing the athletes’ need for quality sleep [74,75,76,77]. Actigraphy based sleep assessments reveal suboptimal sleep in athletes, i.e., low TST and high WASO, causing resultant low sleep efficiency [27,30], which improves following a rest day [78]. However, the athletes’ experience of suboptimal sleep remains unclear as sleep need varies between individuals; some may report poor sleep while objective measures indicate sufficient sleep [32]. Therefore, subjective measures of sleep quality, quantity and timing are a valuable addition to objective sleep assessments. Combined subjective markers of sleep (e.g., TST, time in bed, sleep efficiency, sleep quality and sleep onset latency) can highlight the sleep need and recovery status of athletes and identify areas to be addressed in terms of sleep optimisation. Moreover, the use of subjective measures within an athletic population allows the assessment of large cohorts of athletes that are difficult to access, i.e., elite athletes.

Animal models have demonstrated that nutrients such as glucose, amino acids, sodium, ethanol and caffeine, as well as the timing of meals can affect circadian rhythms [79]. Neurotransmitters such as serotonin, gamma-aminobutyric acid (GABA), orexin, dopamine, melanin-concentrating hormone, galanin, noradrenaline and histamine that are involved in the sleep–wake cycle [80] are affected by nutrition. In terms of recovery, the adaptive response to training is dictated by a number of variables: duration, intensity, frequency and type of exercise, in combination with timing, quality and quantity of nutrition both pre- and post-exercise [81]. Recovery can be maximised by optimal nutrition practices or reduced by suboptimal nutrition practices. Contemporary research has demonstrated the pivotal role of both macronutrient and micronutrient availability in regulating skeletal muscle adaptations to exercise [81,82,83]. It is important to characterise the sleep quality and quantity of sub-elite and elite athletes and recovery practices. This study aimed to investigate: (i) the quality, quantity and timing of sleep among sub-elite and elite athletes; (ii) the recovery/stress balance of sub-elite and elite athletes; and (iii) the supplement use and alcohol intake of sub-elite and elite athletes. This study also aimed to investigate the difference between elite and sub-elite athletes in terms of their subjective sleep, recovery and nutritional practices. It was hypothesised that the sleep, recovery and nutrition practices of elite athletes would be superior to those of sub-elite athletes.

## 2. Materials and Methods

### 2.1. Participants

A sample (*n* = 338) comprising elite (*n* = 115; male *n* = 74 and female *n* = 41) and sub-elite (*n* = 223; male 129 and female 94) athletes were recruited from both Ireland and the United Kingdom (see Table 1). The elite athletes were recruited directly through Sport Ireland and the national governing bodies (NGBs) of each sport within Ireland and the United Kingdom. The sub-elite athletes were recruited via social media and the researcher’s network within high-performance sport. In line with Swann et al. [84], elite athletes were defined as: (a) currently receiving support/funding through the international carding scheme and/or (b) members of a national/professional team or a recruitment/academy squad and/or (c) nationally ranked in their sport. Sub-elite athletes were defined as those competing at a regional, university and/or national level of organised sport that trained and/or competed for a combined minimum of 400 min per week. Athletes, at either level, were excluded if they were (i) aged <18 years, (ii) training and competing for <400 min per week or (iii) reported a sleep disorder.

### 2.2. Procedure

All eligible athletes were invited to take part in an online survey. All procedures were approved by the research ethics committee of the Faculty of Health and Life Sciences, Northumbria University (date of approval 2 July 2019; Submission ID: 17406). After reading the participant information sheet, participants were invited to provide informed consent and then completed an online survey on Qualtrics^xm^ which consisted of a battery of previously validated and reliable widely used questionnaires assessing sleep, recovery and nutritional practices. Following completion of the survey, participants received a debrief sheet with details of how they could contact the researcher if they wished to receive feedback from the survey.

### 2.3. Measures

In the initial section of the survey, the participants completed demographic data. Participants recorded their gender, age, body mass (kg), height (cm), sport, athlete type (elite or sub-elite), phase of season (pre-season, competition or off-season), normal training time (before 8 a.m., 8 a.m. to 5 p.m. and after 5 p.m.) and training/competition duration per week (mins).

#### 2.3.1. EuroQoL (EQ-5D-5L)

The EQ-5D-5L is a self-report measure of health status as defined across five dimensions—mobility, self-care, activity, pain and depression/anxiety—with one question per dimension. Each dimension is scored on a 5-point Likert scale (0 = No problem to 5 = Severe problem) [85]. The EQ-5D-5L also includes a visual analogue scale on which respondents are instructed to rate their perceived current health state (0–100). The EQ-5D-5L has capacity to discriminate between slight, moderate and severe issues within each domain compared to previous versions [86].

#### 2.3.2. Pittsburgh Sleep Quality Index (PSQI)

The PSQI is a self-report measure of sleep quality [62]. The PSQI consists of 19 items grouped into seven component scores (0–3) which are equally weighted. Although overall global scores (GPSQI) are calculated by summing the seven components (range 0–21, with higher scores indicating poorer sleep quality) the component scores provide subscale ratings of: (i) subjective sleep quality, (ii) sleep latency, (iii) TST, (iv) sleep efficiency, (v) sleep disturbances, (vi) use of sleep medication and (vii) daytime dysfunction [63]. Global scores >5 are generally used to indicate poor sleep quality (63). The PSQI has demonstrated a diagnostic sensitivity (89.6%) and specificity (86.5%) in distinguishing between ‘good’ and poor’ sleepers [87]. However, more conservative scores of ≥8 have been used in athletes to indicate poor sleep, potentially due to the increased sleep needs in this population [55]. Although the empirical discussion around the PSQI cut-offs for athletes is ongoing [38,55], given that athletes often strive for marginal gains in their performance, which can be facilitated through optimised sleep, the identification of both ‘poor’ and ‘moderate’ sleep quality is warranted [43], hence the standard cut-off (≥5) was employed.

#### 2.3.3. Epworth Sleepiness Scale (ESS)

The ESS is an eight-item self-report measure of general daytime sleepiness [88]. Respondents report their daytime sleepiness in particular situations on a Likert scale (0 = Would never doze to 4 = High chance of dozing). Scores are summed to yield a global ESS score (0–24). The EES global score is indicative of daytime sleepiness [89]. Higher scores indicate greater sleepiness, scores >10 suggest excessive daytime sleepiness [88]. In general ESS scores are interpreted in terms of daytime sleepiness as follows: 0–5 low normal, 6–10 higher normal, 11–12 mild excessive, 13–15 moderate excessive and 16–24 severe [65].

#### 2.3.4. The Recovery Stress Questionnaire for Athletes (RESTQ Sport)

The RESTQ-Sport is a 52-item self-report measure of general stress and recovery levels of athletes [90]. The RESTQ-Sport consists of seven general stress components with two items per scale (general stress, emotional stress, social stress, conflicts/pressure, fatigue, a lack of energy, and physical complaints), five general recovery components with two items per scale (success, social recovery, physical recovery, general well-being, and sleep quality), three sport-specific stress components with four items per scale (disturbed breaks, burnout/emotional exhaustion, and fitness/injury) and four sport-specific recovery components with four items per scale (fitness/being in shape, burnout/personal accomplishments, self-efficacy, and self-regulation) [90]. Sub-scale item mean scores can be combined to give a total score for each of the four major sub-scales (i.e., general stress, general recovery, sport-specific stress and sport-specific recovery). Each item is scored on a Likert scale (0 = Never to 6 = Always) based on how often the respondent engaged in a specified activity over the previous three days/nights, with a response of 0 indicating never having experienced the feeling and 6 indicating always experiencing the associated feeling. High scores on stress scales indicate a high level of stress, while high scores on the recovery scales indicate a high level of recovery [90].

#### 2.3.5. Athlete Morningness/Eveningness Questionnaire (AMES)

The AMES, which is based on the Horne–Östberg morningness/eveningness questionnaire [91], is a four-item questionnaire used to classify an athlete’s chronotype in terms of self-identification as being a morning or evening type, preferred sleep/wake phase and preferred competition and training time [92]. The AMES provides a global score which is used to categorise chronotype: extreme evening type (10–12), moderate evening type (13–17), mid-range type (18–23), moderate morning type (24–28) and extreme morning type (29–31) [55].

#### 2.3.6. Consensus Sleep Diary—Core (CSD-C)

Participants were instructed to complete the CSD-C for two nights (1 ‘training/competition’ day and 1 ‘rest’ day). The CSD is a standardised sleep diary developed for use in both research and clinical settings [68]. The CSD-C included 8 items, e.g., bed time, time it took to fall asleep, number of awakenings, duration of awakenings, time of final awakening, time the respondent got out of bed, and a Likert scale self-report rating of sleep quality [93]. There was also a comments section where participants could record specific comments about each night’s sleep (i.e., 1 training/competition day and 1 rest day). The data collected were then used to compute indices of sleep continuity such as total time in bed (TIB), total sleep time (TST, sleep onset latency (SOL; time from lights out to N1), wakefulness after sleep onset (WASO; amount of time awake after sleep onset), number of awakenings (NoA) and sleep efficiency (SE; ratio of TST:TIB) [93].

### 2.4. Supplementation

All participants were instructed to complete questions relating to supplement use (name, dose, frequency and reason for use) on both training/competition days and rest days. Athletes also reported their alcohol consumption (number of drinking sessions and unit consumption per session) in the last month prior to completion of the questionnaire.

### 2.5. Data Analysis

All data were analysed using the Statistical Package for the Social Sciences (SPSS Version 25, IBM Corporation) and Jamovi (Version 1.8.16). Frequency distribution and descriptive statistics were used to present findings [94]. All data were presented as the mean ± standard deviation, and/or frequency. The differences between the groups for athlete type were explored using independent-samples t-tests, chi square tests, Mann–Whitney U and one-way ANOVA [94].

## 3. Results

### 3.1. Participant Characteristics

A total of 338 (elite *n* = 115 and sub-elite *n* = 223) athletes were recruited from a variety of team and individual sports (see Table 1 and Table 2.). The sample consisted of both male (*n* = 203; ~60%) and female (*n* = 135; ~40%) athletes.

A chi square analysis demonstrated no significant differences between the groups for gender (X^2^[1, *n* = 338] = 1.72, *p* = 0.189). While there were statistically significant differences between the groups for age (elite 23.44 ± 4.91 years and sub-elite 25.71 ± 6.27 years; *t*(336) = 3.38; *p* = 0.001) and minutes trained per week (elite 801.35 ± 338.81 and sub-elite 610.02 ± 266.90; *t*(336) = −5.68; *p* ≤ 0.001). An independent-samples t-test indicated no significant differences between the groups in terms of height, body mass and normal training time (time of day when training occurred) (see Table 1).

Chi square analyses demonstrated a statistically significant difference between the groups for sport (X^2^[9, *n* = 338] = 1.72, *p* ≤ 0.001). There were no statistically significant differences between the groups for phase of season: pre-season (elite *n* = 31; sub-elite *n* = 57), competition (elite *n* = 65; sub-elite *n* = 115), off-season (elite *n* = 19; sub-elite *n* = 51) (X^2^[2, *n* = 338] = 1.88, *p* = 0.39). There were statistically significant differences between the groups for normal training time: before 8 a.m. (elite *n* = 8 and sub-elite *n* = 25), between 8 a.m. and 5 p.m. (elite *n* = 50; sub-elite *n* = 58), and after 5 p.m. (elite *n* = 57; sub-elite *n* = 140) (X^2^[2, *n* = 338] = 10.9, *p* ≤ 0.001).

#### 3.1.1. EuroQoL

There was no statistically significant difference between the groups for their perceived general health rating (0–100) with the elite athlete group reporting slightly higher levels of general health than the sub-elite athlete group (83.05 ± 13.65 vs. 81.05 ± 12.57; t = −1.37; *p* = 0.172). There were no statistically significant differences between the groups in terms of each of the domains of the quality of life measure (see Table 3). Slight to severe problems with mobility were reported by 19% (*n* = 65) of participants (elite *n* = 19 [17%]; sub-elite *n* = 46 [21%]). Some issues regarding the completion of usual activities (e.g., work, study, training, housework, family or leisure activities) were reported by 19% (*n* = 64) of participants (elite *n* = 23 [20%]; sub-elite *n* = 41 [18%]). Issues with self-care were not evident within the athletes as slight to moderate issues were reported by 3% of participants (elite *n* = 2 [2%]; sub-elite *n* = 9 [4%]). Pain was reported by 50% (*n* = 169) of participants (elite *n* = 53 [46%]; sub-elite *n* = 116 [52%]). Anxiety/depression was reported by 34% (*n* = 116) of participants (elite *n* = 43 [37%]; sub-elite *n* = 73 [33%]).

#### 3.1.2. Pittsburgh Sleep Quality Index

An independent-samples *t*-test was used to compare PSQI data for the elite and sub-elite athlete groups. A statistically significant difference was observed between the groups for PSQI habitual sleep efficiency % (elite 88.62 ± 8.84 vs. sub-elite 86.55 ± 9.09; *t* = −2.01; *p* = 0.046). While no other statistically significant differences were observed, the majority of athletes (64%; *n* = 220) were classified as poor sleepers (i.e., global PSQI score ≥5—elite 64% [*n* = 74]; sub-elite 65% [*n* = 146]). Overall self-reported sleep quality did not reflect this as the athletes rated their sleep quality as very good (elite *n* = 19 [17%]; sub-elite *n* = 45 [20%]), fairly good (elite *n* = 68 [59%]; sub-elite *n* = 123 [55%]), fairly bad (elite *n* = 26 [23%]; sub-elite *n* = 50 [22%]) and poor (elite *n* = 2 [1%]; sub-elite *n* = 5 [2%]). Mean total sleep time (hours) varied between the elite athlete (7.58 ± 1.06; range 5–10 h) and the sub-elite athlete groups (7.35 ± 1.05; range 4–10 h) but this was not statistically significant. The athletes reported total sleep time ≤6 h (elite *n* = 16 [14%]; sub-elite *n* = 43 [19%]), 7 h (elite *n* = 38 [33%]; sub-elite *n* = 80 [36%]), 8 h (elite *n* = 39 [34%]; sub-elite *n* = 70 [32%]) and 9 h (elite *n* = 22 [19%]; sub-elite *n* = 30 [13%]). The athletes’ responses to the PSQI are summarised in Table 3. The athletes reported total time in bed 8 h (elite *n* = 53 [46%]; sub-elite *n* = 109 [49%]), 9–10 h (elite *n* = 50 [44%]; sub-elite *n* = 110 [49%]) and 11–12 h (elite *n* = 12 [10%]; sub-elite *n* = 4 [2%]).

The reasons reported for poor sleep quality were not getting to sleep within 30 min, waking during the night or early morning, waking to use the bathroom and feeling too hot in bed (see Table 3). The feeling of a lack of enthusiasm for general tasks at least once per week was reported by 44% (*n* = 51) of the elite group and 41% (*n* = 92) of the sub-elite group. The use of sleep medication was low in both groups, with 5% (*n* = 6) of the elite group and 7% (*n* = 16) of the sub-elite group using medication on a weekly basis (see Table 4).

### 3.2. Epworth Sleepiness Scale

An independent-samples *t*-test demonstrated no significant differences between the elite and sub-elite athlete groups for ESS scores (*p* > 0.05). A chi square test highlighted no significant difference between the groups’ ESS classification (X2[20, *n* = 338] = 21.1, *p* = 0.391). Approximately 21% (*n* = 70) of athletes (elite *n* = 25; 22% and sub-elite *n* = 45; 20%) reported clinically significant excessive daytime sleepiness (ESS total score ≥10) (see Table 5).

### 3.3. Recovery Stress Questionnaire

An independent-samples *t*-test highlighted significant differences between the elite and sub-elite athlete groups for recovery, i.e., the sport-specific recovery scale (3.22 ± 0.90 vs. 2.91 ± 0.90; *t* (−2.984); *p* < 0.001). While no statistically significant differences were observed for the general stress, general recovery and sport-specific stress subscales. Recovery stress scale scores were similar in both the elite and sub-elite groups with similar scores observed for the general stress scale (1.96 ± 0.91 vs. 2.01 ± 0.86), general recovery scale (2.97 ± 0.79 vs. 2.97 ± 0.77) and sport-specific stress scale (1.97 ± 0.87 vs. 1.99 ± 0.85).

**Figure 1:**
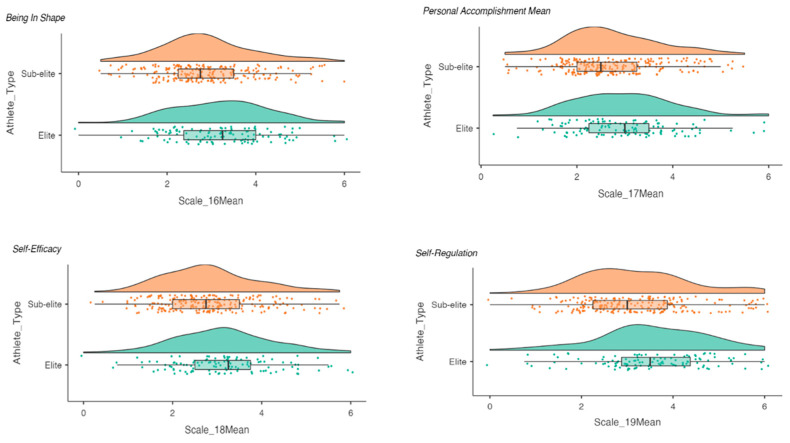
Comparison of the sport-specific recovery subscales.

An independent-samples *t*-test displayed no statistically significant differences between the groups for the majority of the subscales with both groups recording similar scores (see Table 6). However, significant differences between the groups were observed for the following sport-specific recovery subscales: being in shape (3.22 ± 1.08 vs. 2.90 ± 1.04; *t =* −2.66; *p* = 0.008), personal accomplishment (2.97 ± 1.04 vs. 2.74 ± 0.98; *t* = −1.98; *p* = 0.048), self-efficacy (3.15 ± 1.12 vs. 2.83 ± 1.04; *t* = −2.58; *p* = 0.010) and self-regulation (3.55 ± 1.19 vs. 3.18 ± 1.18; *t* = −2.71; *p* = 0.007), with higher levels being observed across each domain in the elite athlete group (see Figure 1). While not statistically significant poor sleep quality was observed (2.77 ± 0.78 vs. 2.83 ± 0.85), concerns related to injury (2.48 ± 1.09 vs. 2.32 ± 1.17) and relatively high levels of fatigue (2.46 ± 1.33 vs. 2.54 ± 1.31).

#### 3.3.1. AMES

An independent-samples *t*-test demonstrated a statistically significant difference between the groups for preferred competition time (*t* (336) = −2.45; *p* = 0.015), with a higher percentage of the elite athlete group (77% [*n* = 89]) preferring afternoon competition times compared to the sub-elite group (60% [*n* = 113]) (see Table 7). There was no significant difference between the groups for chronotype, time they usually become tired and preferred training time.

#### 3.3.2. Consensus Sleep Diary—Core

All athletes also completed a sleep diary for a training/competition day and a rest day. A one-way ANOVA was conducted to assess the difference between the groups for TIB, TST, SL, NoA and WASO on both the training/competition day and rest day. While there were no statistically significant differences for TIB, SL and WASO, there were statistically significant differences between the groups (elite vs. sub-elite) for TST on the training/competition day (8.01 ± 1.3 vs. 8.2 ± 1.38; F(1, 238) = 3.91; *p* = 0.049) and NoA on the rest day (1.03 ± 1.17 vs. 1.52 ± 2.44; *F*(1, 334) = 6.34; *p* = 0.012), with the sub-elite athlete group reporting higher levels of both measures (see Table 8). The majority of athletes in both groups (elite *n* = 155 [70%]; sub-elite *n* = 77 [67%]) reported wakening 1–5 times each night. Athletes in both groups reported that it took ≥30 min to fall asleep on the training/competition day (elite *n* = 33 [29%]; sub-elite *n* = 72 [32%]) and the rest day (elite *n* = 35 [30%]; sub-elite *n* = 70 [31%]). While there was no statistically significant difference between the groups, poor habitual sleep efficiency (<85%) was reported by 20% (*n* = 23) of the elite athlete group and 25% (*n* = 55) of the sub-elite athlete group. In the comments section of the sleep diary a subset of athletes (*n* = 73 [22%]) reported the reasons for waking at night, the most common reasons included injury (*n* = 15 [4%]), children (*n* = 11 [3%]), anxiety (*n* = 19 [6%]), energy restriction (i.e., making weight) (*n* = 7 [2%]) and waking to use the bathroom (*n* = 21 [6%]).

#### 3.3.3. Nutrition

The athletes also reported their supplement and alcohol consumption in the month prior to completion of the questionnaire. A Mann–Whitney U test indicated no significant differences between the elite and sub-elite athlete groups for supplementation and alcohol consumption (*p* ≥ 0.05). The most commonly used supplements were whey protein, caffeine, creatine, multivitamins, fish oil, probiotics and vitamin D (see Table 9).

Spearman’s rank order correlation was used to assess the relationship between supplement use and various sleep and recovery variables. There were small significant correlations between supplement use and the RESTQ scales: sleep quality, disturbed breaks, emotional exhaustion, being in shape and self-efficacy (see Table 10).

The athletes reported the number of times that they consumed alcohol in the last month 1–4 times (elite *n* = 10 [9%]; sub-elite *n* = 10 [5%]), 5–9 times (elite *n* = 11 [10%]; sub-elite *n* = 5 [2%]), and >10 times (elite *n* = 3 [3%]; sub-elite *n* = 11 [5%]). The athletes also reported the number of units they usually consumed during each drinking session <4 units (elite *n* = 11 [10%]; sub-elite *n* = 6 [3%]), 5–10 (elite *n* = 9 [8%]; sub-elite *n* = 8 [4%]) and >10 (elite *n* = 4 [3%]; sub-elite *n* = 12 [5%]).

## 4. Discussion

This study recruited a large cohort of elite (*n* = 115) and sub-elite (*n* = 223) athletes from a wide variety of sports. Elite athletes were either international athletes, members of a national/professional team, a recruitment/academy squad and/or nationally ranked in their sport [84]. Sub-elite athletes were defined as those competing at a regional, university and/or national level of organised sport that trained and/or competed for a combined minimum of 400 min per week [84]. To the authors’ knowledge, this is one of the largest cohorts of athletes to have been investigated from a sleep and recovery perspective. This study aimed to investigate: the quality, quantity and timing of sleep among sub-elite and elite athletes and characterise their recovery and nutrition practices. It was hypothesised that the sleep, recovery and nutrition practices of elite athletes would be superior to those of sub-elite athletes. Interestingly, similar levels of poor sleep were reported by both the elite and sub-elite athlete groups, whereas there was a significant difference in sport-specific recovery practices.

### 4.1. Sleep

Poor sleep quality was reported in the PSQI, the REST-Q and it was notable in the sleep diaries that athletes reported improved TIB, TST and WASO on rest days. Excessive daytime sleepiness was also observed in both groups. Similarly, previous research has suggested that the quality and quantity of elite athlete’s sleep was inferior to sub-elite athletes and potentially inadequate in relation to optimal recovery and performance [27,30,32,37,95].

### 4.2. Pittsburg Sleep Quality Index

The PSQI has demonstrated good reliability (Cronbach’s alpha = 0.83, test–retest reliability *r* = 0.85) [87]. The PSQI having demonstrated acceptable internal consistency and has been shown to be reliable [96,97] and valid [87,96,97,98] measure of sleep quality. Cronbach’s alpha 0.744 was observed in the current sample. The majority of athletes (~65%; *n* = 220) were classified as poor sleepers (Global PSQI score ≥5). This is consistent with previous research in elite athletes [53,54,55,95], and sub-elite athletes [99,100]. A relatively high proportion of athletes (~30%) self-reported their sleep quality as either poor or very poor on the training/competition day compared to rest day (elite 10% [*n* = 12] and sub-elite 16% [*n* = 36]). The PSQI data highlighted reasons for poor sleep on both training/competition days and rest days such as feeling too hot in bed and a lack of enthusiasm for general tasks. Poor sleep quality is of particular concern for elite athletes as it can result in a reduction in recovery and/or subsequent athletic performance [29,101,102,103].

Interestingly the PSQI mean TST (<8 h) was lower than that reported in the CSD-C (>8 h), it has been suggested that athletes tend to overestimate their sleep [104,105]. A recent review suggested that sleep in athletes is limited to 7.2 h per night, with all studies reporting <8 h per night and mean SE was 86.3 ± 6.8% [106], which is in line with the PSQI and CSD-C data from the current study. The PSQI mean TST for both groups in the current study is adequate according to current sleep recommendations (7–9 h) [48]. However, optimal TST is subject to individual variance and it has been argued that elite athletes may require more quality sleep than non-athletes [49]. It has previously been reported that athletes tend to sleep less (6.5–6.7 h) and that their sleep quality is poor [27,54,107,108,109]. Optimising sleep gives athletes an advantage when it comes to maximising adaptations from training and performance enhancement [110].

### 4.3. Consensus Sleep Diary-Core

There were significant differences between the groups for TST on the training/competition day and NoA on the rest day. TST was lower in the elite athlete group on both days. However, it did improve on the rest day which was most likely a reflection of their behaviour, e.g., choosing to go to bed earlier. Although not statistically significant there was a trend towards reduced TIB, TST and WASO in both groups on the rest day while the elite athlete group also demonstrated a trend towards reduced SL, NoA and increased SE on the rest day. Similarly, a small study of Australian athletes (*n* = 6) using objective measures of sleep demonstrated that sleep improved (longer duration) on a rest day (71.6% reported no sleep disturbance following one rest day) [78]. A study involving elite swimmers (*n* = 7) showed that the athletes went to bed later but slept longer on rest days [54], where the opportunity for extended sleep provided the athletes with an opportunity to partially recover the sleep debt accumulated during the training week [111]. In the current study, poor sleep was attributed by the athletes in both groups to a number of factors, i.e., injury, children, anxiety, making weight (boxing) and bathroom use. Previous research has highlighted issues that impair an athlete’s sleep such as stress [32,112], pain/injury [26,32,33] and anxiety [25,29]. The relationship between poor sleep and impaired mood has been reported in non-athletic populations [113]. However, the study involved sleep restriction to 4.98 h per night. Monitoring athletes’ mood (e.g., through wellness monitoring) could identify athletes who require sleep-related intervention.

In the current study, poor habitual SE% previously quantified as <85% [47] was reported by 20% (*n* = 23) of the elite athlete group and 25% (*n* = 55) of the sub-elite athlete group. Previous research has demonstrated that habitual sleep efficiency of elite athletes was 88.47 ± 5.45% [95] 80.6 ± 6.4% [27], 86.3 ± 6.1% [30] and 79 ± 9.2% [114]. A recent systematic review reported the pooled average sleep efficiency for athletes (86 ± 5%; range 79–96%) [37] which straddled and for many athletes overlapped the threshold of 85%, below which insomnia symptoms are indicated [115]. While the range of sleep efficiency observed can in part be explained by methodological inconsistencies, the pooled mean nonetheless indicated sleep problems and poor sleep quality. There is a need for clear athlete-friendly interventions that could promote improved sleep and recovery.

#### 4.3.1. Daytime Sleepiness

The ESS score is comparable to objective sleepiness measures such as the multiple sleep latency test (MSLT) and is considered a valid and reliable measure of objective sleepiness [88]. The ESS has been widely used in athletic populations such as Australian rules football [116], collegiate basketball players [109] and American football players [117]. In the present sample, Cronbach’s alpha was 0.827. Approximately 21% of athletes in the current study reported excessive daytime sleepiness. Similar levels of excessive daytime sleepiness have been reported in rugby players and cricketers [53], American footballers [117], Australian rules footballers [116] and college athletes [99]. Similarly, previous research reported that 44% (*n* = 12) Brazilian Paralympians experienced excessive daytime sleepiness [118]. However, it must be noted that these athletes may have had physical impairments (e.g., spinal cord injury) that could impact sleep quantity and quality.

The levels of excessive daytime sleepiness observed in the current study may be due to sleep disorders such as obstructive sleep apnoea (OSA) and periodic limb movement disorder (PLMD). In the general population, the most common sleep disorders are (OSA), insomnia and restless legs syndrome (RLS)/(PLMD) [61]. OSA is a frequent condition characterised by repeated episodes of partial or complete reduction in breathing activity during sleep [61]. PLMD is a condition characterised by repetitive limb movements during sleep that cause sleep disruption [62]. A recent systematic review highlighted the prevalence of insomnia symptoms (longer SOL, increased sleep fragmentation and excessive daytime sleepiness) in elite athletes [37]. Other sleep problems such as OSA are less prevalent but appear to higher in strength and power athletes (e.g., rugby players) most likely due to increased body mass and neck circumference (>42 cm) which are anatomical features related to OSA [53]. A recent study using a combination of PSG and subjective measures demonstrated a high prevalence of sleep disorders in Rugby union players (*n* = 25), all players displayed insomnia symptoms and 24% (*n* = 6) had OSA and 12% (*n* = 3) [64]. In similar study using home-based PSG in rugby league players (*n* = 22), 45% (*n* = 10) had OSA [119]. A previous study of NFL players (*n* = 137) demonstrated that 19% (*n* = 26) had OSA [120]. Previous research in elite ice hockey players (*n* = 107) has demonstrated sleep problem, 11% (*n* = 14) had insomnia, 10% (*n* = 13) had OSA and 3% (*n* = 4) had RLS/PLMD [26]. Athletes with poor sleep habits and/or a sleep disorder must be identified and diagnosed and individual interventions (e.g., sleep hygiene, nutrition) must be implemented in order to athlete recovery and performance.

#### 4.3.2. Athlete Morningness/Eveningness

A Cronbach’s alpha of 0.698 was observed in the current sample. Although there was no significant difference between the groups for chronotype, time they usually become tired or preferred training time, a statistically significant difference was evident for preferred competition time, (*p* = 0.015), with the elite athlete group preferring afternoon competition times, while the sub-elite athlete group preferred morning competition times. The vast majority of the athletes from both groups 58% (*n* = 197) indicated that their normal training time was after 5 p.m. Training time and chronotype may have an influence on sleep [40]. A study investigating the sleep quality of morning and evening types after a morning (8:00 a.m.) and evening (20:00 p.m.) high intensity interval training session types reported poorer sleep quality (reduced total sleep time, increased sleep disturbance and reduced sleep efficiency) in morning types after the evening session while sleep quality after the morning session was similar for both groups [121]. The late training times reported by the athletes in the current study may have adversely impacted their sleep and recovery. Sleep following training is recognised a being important for recovery [122], reduced sleep quality following evening training sessions (particularly vigorous training) may negatively impact subsequent recovery and performance, the effect may be more pronounced in morning type athletes.

### 4.4. Recovery

Recovery is a process in time, dependent on the duration of stress and requires a reduction in stress, a change in stress or a break from stress [123,124]. Relatively high levels of fatigue, stress and pain were reported in both groups. A range of supplements were used regularly by athletes in both groups; indeed, whey was the most commonly used recovery supplement in both groups. The results suggest that future research is warranted to further the development of individualised inventions focused on sleep, nutrition and athlete recovery.

#### 4.4.1. EuroQoL

The EQ-5D-5L has demonstrated reliability (mean intraclass correlation coefficients 0.69; range 0.43–0.84) and convergent validity (mean Spearman rank coefficients 0.99; range 0.97–0.99) [85]. Cronbach’s alpha of 0.70–0.95 are considered “acceptable” for a scale used in human research [125,126]. Cronbach’s alpha 0.609 was observed in the current sample most likely due to the low number of items (5), as the size if alpha depends on the number of items in a scale [127]. The mean general health rating scores for the elite athlete group (83.1 ± 12.6) and the sub-elite athlete group (81 ± 13.7) were relatively high, which was consistent with current research in athletes [128]. In the current study, the elite athlete group reported higher mean health rating scores. Elite athletes tend to have their training and recovery sessions scheduled for them [54], hence, they are likely to complete regular if not daily mobility type sessions. Whereas the sub-elite athletes may have had less free time due to work, social and family commitments. A high prevalence of pain was reported by 50% (*n* = 169) of participants (elite *n* = 53 sub-elite *n* = 116). An investigation of ‘mildly sleepy’ (indicative of inadequate TST) but otherwise healthy males (*n* = 24) showed sleep extension (time in bed 10 h) increased pain tolerance by 20% [129]. While chronic sleep restriction (50% of habitual time for 12 days) is related to increased levels of muscle soreness and increased pain sensitivity [107]. While mobility issues were noted in both groups, there were higher levels mobility issues reported by the sub-elite athlete group coupled with issues completing usual activities. However, it has recently been suggested that elite and high-level athletes have increased pain tolerance (cold pressor test) and that the training time per week has a positive impact on the tolerance [130].

#### 4.4.2. REST-Q Sport

The RESTQ-Sport has been shown to be valid in athletic populations [131,132]. The scales have displayed good internal consistency (0.67–0.89) and high test–retest reliability (>0.79) [90,124]. A Cronbach’s alpha of 0.784 was observed in the current sample.

Relatively high levels of stress and fatigue were evident from the REST-Q. Stress and fatigue are factors for illness, which must be managed by elite athletes [133,134], during their competitive seasons to avoid missed training/competitions. Significant differences between the elite and sub-elite athletes were observed for four of the REST-Q subscales relating to athletic performance, with higher mean score for each subscale: being in shape, personal accomplishment, self-efficacy and self-regulation reported by the elite athlete group. The injury (2.31 ± 1.17 vs. 2.48 ± 1.09), fatigue (2.45 ± 1.32 vs. 2.54 ± 1.32) subscale scores were relatively high in both the elite athletes and sub-elite athletes, while the sleep quality scores were low (2.76 ± 0.78 vs. 2.83 ± 0.85). The current findings are consistent with previous research which reported that injury risk was significantly positively related to injury subscale scores for disturbed breaks, fatigue, and lower values on the sleep quality subscale score [131]. The relationship between training load and health can be considered on a well-being continuum [123,134,135], with training load and recovery as antagonists. Stress is imposed on athletes, altering their physical and psychological well-being along a continuum: homeostasis, acute fatigue, subclinical tissue damage, functional overreaching, non-functional overreaching, clinical symptoms, overtraining syndrome, time-loss injury or illness and, with continued loading in extreme cases, death [134,135]. A recent meta-analysis has linked psychological stress (*r* = 0.27, 80% CI 0.20–0.37) and history of stressors (*r* = 0.13, 80% CI 0.11–0.15) to injury rates [136]. Athletes’ injury risks are affected by their responses to multiple stressors that result in not only physical, psychological and attentional changes (e.g., increased reaction time, narrowing of peripheral vision, increased distraction) but also behavioural changes (e.g., poor sleep quality and impaired self-care) [136].

In the current study, significantly higher levels of sport-specific recovery (3.22 ± 0.91 vs. 2.91 ± 0.90) were reported by the elite athlete group compared to the sub-elite athlete group. This result potentially highlights the fact that elite athletes tend to be under the supervision of a multidisciplinary team, e.g., medical, strength and conditioning, nutrition, physiology and psychology, who are involved in all aspects of the athletes training and recovery. The sub-elite athletes would typically not receive the same access to multidisciplinary support services. It is imperative that athletes have a detailed recovery plan compromising of nutrition, hydration, sleep and psychological recovery [134]. Given the high training and competition load that athletes undertake, it is clear that they must adopt strategies that promote sleep across the domains of quality, quantity and timing. Fatigue can be managed, and recovery enhanced through adequate passive rest and sufficient sleep [137], it is generally recommended that athletes have at least one ‘rest’ day per week. Rest days can serve to alleviate boredom and stress perception while the absence of a ‘rest day’ during periods of intense training has been related to the onset overreaching and inadequate recovery [137]. It is suggested from the current results that sleep tends to improve on rest days, i.e., increased perceived sleep quality, TIB, TST and reduced WASO in both groups, while SL, NOA and SE also improved in the elite athlete group.

### 4.5. Nutrition

In the current sample, the elite athletes tended to consume more supplements, at higher doses with increased frequency, compared to the sub-elite athletes. Those athletes who used supplements reported high usage of caffeine, whey protein, creatine, multivitamins, fish oil, probiotics and vitamin D while the use of iron and nitrate was reported to a lesser extent. This is similar to previous research in elite Dutch athletes (*n* = 778) where the most commonly consumed supplements were multivitamins, caffeine, vitamin D, sports drinks, protein, beta-alanine and sodium bicarbonate [138]. It has also been demonstrated previously that elite athletes tend to take more supplements than sub-elite athletes [139]. Despite the relatively low number of athletes reporting supplement use, the correlations between supplement use and RESTQ scales warrant further investigation. Whey protein was one of the most prevalent supplements used while casein use was also reported. While research is emerging supporting pre-sleep protein ingestion for muscle recovery [140,141], the impact of pre-sleep ingestion of 40 g doses of whey and/or casein warrants further investigation with regards both muscle recovery and sleep improvement.

Daily caffeine use was reported by approximately 20% of the athletes which could negatively impact sleep. The low level of caffeine use reported in the current study was most likely due to the fact that athletes were asked to report their supplement use and may have neglected to include habitual caffeine consumption. Caffeine exerts a stimulant effect promoting alertness by blocking adenosine receptors [142]. The levels of caffeine consumption reported were lower than previous research which has suggested that 75–90% of athletes consume caffeine before or during competition [143,144,145]. While, it has been suggested that chronic low dose caffeine ingestion may blunt any potential ergogenic effects [146], moderate doses (~3 mg/kg/d) appear to pose no problems for most athletes [147]. However, in terms of sleep, moderate caffeine doses have been shown to increase SOL and decrease TST, REM sleep and SE [148]. Hence, athletes training/competing in the late afternoon (>5 p.m.) need to consider its potentially detrimental effect on sleep. It has recently been suggested that athletes should adopt a strategic individualised approach to caffeine consumption during competition [149]. In the current study, higher alcohol consumption was observed in the sub-elite athletes and they tended to consume more units of alcohol per drinking bout. In line with previous research, the actual amount of alcohol consumed by athletes “in training’ is low [150]. Elite athletes tend to have less opportunity to socialise and their schedules (e.g., early morning training) do not lend themselves to regularly consuming alcohol. Alcohol consumption by athletes often occurs post-competition, where it can be seen as a reward for ‘hard work’ [151]. Alcohol consumption has been associated with poorer sleep quality and quantity, reduced REM sleep and increased sleep disturbance in the second half of the sleep bout [152].

### 4.6. Limitations

Due to logistical reasons, the sleep diary was only completed for one training/competition day and one rest day, and this may have been insufficient in terms of data collection. It has been recommended that sleep diaries should be completed for a duration of 1 week [68,153]. The aim of the 2 day diary was to limit participant burden and recall bias [154]. However, sleep diaries may be more accurate than sleep questionnaires [32]. The intrinsic limitations of self-report measures (i.e., questionnaires and diaries) are measurement error and recall bias [94]. Indeed, it has been demonstrated that athletes can overestimate their TST [104,105]. However, self-report measures have their place within athletic settings, as they are a relatively simple and inexpensive approach to athlete monitoring affording a more representative overview of the target population [44]. Within elite athlete populations, the use of subjective measures of sleep are often employed, particularly during the competitive season due to the more invasive nature of both PSG and actigraphy [12]. A growing body of research has suggested that self-report measures may be more sensitive and reliable than physiological, biochemical and performance measures [44,137,153,154,155,156]. When choosing a particular measure, ultimately the aim is to maintain a balance between the need to obtain meaningful data from an athlete whilst minimising the burden involved in completion of any self-report measure [154,155,156]. In the current study, it was not feasible or practical due to the large sample size to include a subjective assessment of sleep. However, future research should incorporate both objective (e.g., PSG, actigraphy) and subjective measures (e.g., sleep diaries) of sleep to provide a more accurate estimates of sleep and because some individuals may self-report poor sleep quality despite objective measures indicating adequate sleep [155,156,157]. There was little difference between the elite and sub-elite athlete groups in terms of sleep. The inclusion of a healthy control group would have allowed for comparison and exploration of the differences between the sleep of athletic population and healthy adults.

A specific section in relation to anxiety/depression could have been included in the battery of questionnaires given the potential to impact on sleep and vice versa. The Profile of Mood States (POMS) [158] is widely used in wellness assessments of athletic populations and has subscales that specifically relate to anxiety and depression. However, as the EuroQoL has a dimension for anxiety/depression, the POMS was omitted to reduce participant burden and survey fatigue which could have negatively impacted the reliability of the data collected.

The demographic difference between the groups was a limitation in that there was a statistically significant difference between the groups with the sub-elite group being significantly older which could have affected the results. This issue was directly related to the sampling method employed where participants are recruited based on their accessibility. However, care was taken to recruit a large cohort (*n* = 338) and strict inclusion and exclusion criteria were applied [84].

### 4.7. Future Research

Future research should replicate this investigation of the sleep and recovery practices of large cohorts of athletes. Such studies should include a combination of subjective and objective measures of sleep and recovery, for a minimum of 1 week [54,153]. The validity and reliability of combinations of subjective and objective measures in athletic populations warrants further investigation. While this may not be practical during the competitive season there may be a window of opportunity at the end of the season or in preseason.

As the majority of athletes in the current cohort have reported sleep problems future research is warranted to identify the specific sleep problems that affect athletic populations. It is also necessary in future research to identify if athletes are affected by acute disturbances, e.g., competition anxiety or chronic disorders, e.g., OSA, insomnia and PLMD [26].

Future research should investigate the effects of specific nutritional recovery strategies (e.g., antioxidants, protein, carbohydrate) on sleep in athletic populations. Such practices may already be an established part of an athlete’s daily routine, but the potential additional benefit of improved sleep must be explored.

### 4.8. Practical Applications

A strength of this novel study is that it presents ‘real-life’ data from training/competition days and a rest day relating to the sleep and recovery practices of athletes. Poor sleep and inadequate recovery practices were evident in both the elite and sub-elite athlete groups. In a recent study, 95% of swimmers (*n* = 82) identified their coaches (*n* = 10) as the primary source of recovery information while the coaches highlighted conferences and workshops as their primary source of recovery information [159]. In order to promote sleep hygiene and adequate recovery practices in athletes, a comprehensive coach and athlete education curriculum may need to be developed and implemented.

The athletes generally reported improved sleep quality and quantity on rest days which has implications for athlete health, well-being and performance. Optimising the sleep and recovery practices of athletes would impact performance. Monitoring of sleep behaviours, nutrition and recovery-stress responses of athletes aids the identification of irregularities (e.g., due to travel or illness) and allows for early interventions with individual athletes as and when necessary [157]. The ongoing collection of data from athletes such as the data collected in the current study could be used by coaches and medical and support staff to implement individual sleep, recovery and nutrition interventions and plans.

## 5. Conclusions

Due to the symbiosis between sleep and recovery, it is clear from the current findings that athletes should have a detailed individualised and multifaceted recovery plan in place involving sleep, nutrition, hydration, and other physiological and psychological aspects. At the elite level, athletes and their support teams continually strive for marginal gains over time to improve performance (135). Training and competition load elicit a number of homeostatic responses and adaptations, and the main aim of training is to exploit these in order to elicit an improvement in performance. The training process involves exploitation, manipulation and coordination of numerous variables (e.g., physiology, biomechanics and psychology) to improve performance. Athletes continually strive to improve their performance, and, as such, variations in training load are necessary, e.g., increased frequency, duration and/or intensity in order to optimise the training response [44]. Depending on the phase of the season (e.g., pre-season, general preparation, and competition), loads must be managed to increase or decrease fatigue, to enhance training adaptations or performance [44]. Rest days should also be incorporated into the recovery plan, which could serve to improve sleep quality, alleviate boredom and stress perception.

The majority of athletes were classified as poor sleepers and reported excessive daytime sleepiness even though their TST met current adequate sleep guidelines. The importance of a rest day was highlighted by the fact that sleep improved in both groups. Relatively low levels of physical recovery were observed in both groups coupled with relatively high levels of stress. The elite athlete group reported significantly higher levels of sport-specific recovery. A higher prevalence of supplement use was reported by the elite athlete group, while higher levels of alcohol consumption were reported by the sub-elite athlete group. Given the high training and competition load that athletes undertake, particularly elite athletes, it is clear that they must adopt strategies that promote sleep and recovery. There is a need for athletes to receive individualised support and education regarding their sleep ad recovery practices.

## Figures and Tables

**Table 1:** Participant characteristics (mean ± SD).

	All (*n* = 338)	Elite (*n* = 115)	Sub-Elite (*n* = 223)	t/x^2^ Value
Gender	Male *n* = 203; Female *n* = 135	Male *n* = 74; Female *n* = 41	Male *n* = 129; Female *n* = 94	X ^2^ = 1.72
Age *	24.94 ± 5.93	23.44 ± 4.91	25.71 ± 6.27	*t* = 3.384
Body mass (kg)	72.95 ± 13.26	73.95 ± 12.55	72.44 ± 13.61	*t* = −0.995
Height (cm)	175.60 ± 9.70	176.6 ± 8.78	175.08 ± 10.12	*t* = −1.361
Training (mins·wk) *	675.12 ± 306.59	801.35 ± 338.81	610.02 ± 266.90	*t* = −5.682

* Statistically significant difference.

**Table 2:** Participant breakdown.

Sport	All	Elite *n* = 115	Sub-Elite *n* = 223
Athletics	64	10	54
Boxing	12	11	1
Gaelic games	89	26	63
Hockey	10	9	1
Rowing	29	8	21
Rugby	20	8	12
Sailing	4	3	1
Soccer	31	10	21
Swimming	8	4	4
Other	71	26	45

**Table 3:** Athlete responses to the EuroQOL.

		None	Slight	Moderate	Severe	Extreme
Mobility	Elite	96	14	5		
	Sub-elite	177	42	2	1	1
Self-care	Elite	113	1	1		
	Sub-elite	214	7	2		
Usual activities	Elite	92	18	3	1	1
	Sub-elite	182	33	8		
Pain	Elite	62	47	6		
	Sub-elite	107	102	14		
Anxiety/Depression	Elite	72	33	8	2	
	Sub-elite	150	58	13	2	

**Table 4:** Athlete responses to the PSQI.

		Not during the Last Month	Less than Once per Week	Once or Twice per Week	Three or More Times per Week
Cannot get to sleep within 30 min	Elite Sub-elite	40 99	24 56	27 82	34 48
Wake up in the middle of the night or early morning	Elite Sub-elite	30 37	24 56	27 82	34 48
Have to get up to use the bathroom	Elite Sub-elite	38 63	24 76	29 44	24 40
Cannot breathe comfortably	Elite Sub-elite	101 192	11 16	1 10	2 5
Cough or snore loudly	Elite Sub-elite	88 167	14 33	5 16	8 7
Feel too cold	Elite Sub-elite	79 160	27 41	7 19	2 3
Feel too hot	Elite Sub-elite	54 82	32 77	26 54	3 10
Have bad dreams	Elite Sub-elite	63 114	34 75	16 27	2 7
Have pain	Elite Sub-elite	81 152	22 48	11 19	1 4
Other reasons	Elite Sub-elite	101 180	9 27	3 9	2 7
Problems staying awake	Elite Sub-elite	66 125	29 66	13 27	7 5
Lack of enthusiasm	Elite Sub-elite	35 50	29 81	37 69	14 23
Use of sleep medication	Elite Sub-elite	104 189	5 18	3 7	3 9

**Table 5:** ESS classification.

Classification (ESS Score)	Elite (*n* = 115)	Sub-Elite (*n* = 223)
Low Normal (0–5)	53	114
Higher Normal (6–10)	45	70
Mild Excessive (11–12)	6	20
Moderate Excessive (13–15)	8	14
Severe (16–24)	3	5

**Table 6:** RESTQ scales (Mean ± SD).

	All (*n* = 338)	Elite (*n* = 115)	Sub-Elite (*n* = 223)	T=	*p*=
General Stress	1.7 ± 1.31	1.77 ± 1.39	1.67 ± 1.26	−0.6602	0.51
Emotional Stress	1.95 ± 0.983	1.9 ± 0.98	1.97 ± 0.99	0.6858	0.493
Social Stress	1.85 ± 1.03	1.83 ± 1.04	1.86 ± 1.02	0.2199	0.826
Conflicts/Pressure	2.35 ± 1.24	2.24 ± 1.26	2.41 ± 1.24	1.1382	0.256
Fatigue	2.52 ± 1.32	2.46 ± 1.32	2.55 ± 1.32	0.6125	0.541
Lack of Energy	2 ± 1.06	1.95 ± 1.19	2.02 ± 1	0.5755	0.565
Physical Complaints	1.61 ± 1.22	1.59 ± 1.34	1.61 ± 1.16	0.1638	0.87
Success	2.85 ± 1	2.92 ± 1.01	2.81 ± 1	−0.9189	0.359
Social Relaxation	3.3 ± 1.28	3.19 ± 1.26	3.36 ± 1.29	1.1573	0.248
Physical Relaxation	2.53 ± 1.06	2.59 ± 1.09	2.49 ± 1.04	−0.8265	0.409
General Well-Being	3.35 ± 1.16	3.37 ± 1.22	3.35 ± 1.13	−0.1497	0.881
Sleep Quality	2.81 ± 0.83	2.77 ± 0.78	2.83 ± 0.85	0.6552	0.513
Disturbed Breaks	1.68 ± 0.92	1.71 ± 0.91	1.67 ± 0.94	−0.4119	0.681
Burnout/Emotional Exhaustion	1.83 ± 1.13	1.87 ± 1.22	1.81 ± 1.09	−0.4695	0.639
Fitness/Injury	2.43 ± 1.12	2.32 ± 1.17	2.48 ± 1.09	1.2827	0.2
Fitness/Being in Shape **	3.01 ± 1.06	3.22 ± 1.08	2.9 ± 1.04	−2.6563	0.008
Burnout/Personal Accomplishment *	2.82 ± 1.01	2.97 ± 1.04	2.74 ± 0.98	−1.9984	0.048
Self-Efficacy **	2.94 ± 1.07	3.15 ± 1.12	2.83 ± 1.04	−2.5747	0.01
Self-Regulation **	3.31 ± 1.2	3.55 ± 1.19	3.18 ± 1.18	−2.7121	0.007

Data presented as the mean ± SD * *p* < 0.05, ** *p* < 0.01.

**Table 7:** Athlete response to the AMES.

Chronotype Elite (*n* =) Sub-elite (*n* =)	Morning type	More morning type	More evening type		Evening type
	24 45	40 84	36 65		15 29
Preferred training time	6 a.m.–9 a.m.	9 a.m.–Noon	Noon–3 p.m.	3 p.m.–6 p.m.	6 p.m.–9 p.m.
Elite (*n* =) Sub-elite (*n* =)	12 26	31 75	29 47	18 41	25 34
Preferred competition time * Elite (*n* =) Sub-elite (*n* =)	6 a.m.–9 a.m. 5 12	9 a.m.–Noon 21 78	Noon–3 p.m. 47 62	3 p.m.–6 p.m. 21 47	6 p.m.–9 p.m. 21 24
Time you usually get tired	8 p.m.–9:30 p.m.	9:31 p.m.–10:45 p.m.	10:46 p.m.–12:30 a.m.	12:30 a.m.–1:45 a.m.	1:46 a.m.–3:00 a.m.
Elite (*n* =) Sub-elite (*n* =)	27 50	51 94	26 66	3 9	8 4

* Statistically significant difference (*p* < 0.05).

**Table 8:** Sleep diary responses (mean ± SD).

Sleep Measure		Training/Competition Day	Rest Day
TIB (h)	Elite Sub-elite	9.1 ± 1.18 9.2 ± 1.42	9.53 ± 1.49 9.6 ± 1.5
TST (h)	Elite Sub-elite	8.01 ± 1.30 * 8.2 ± 1.38 *	8.58 ± 1.4 8.59 ± 1.44
SL (Min)	Elite Sub-elite	22.85 ± 20.74 22.65 ± 17.70	21.62 ± 18.7 23.72 ± 22.37
NoA (#)	Elite Sub-elite	1.38 ± 1.43 1.51 ± 1.73	1.03 ± 1.17 * 1.52 ± 2.44 *
WASO (Min)	Elite Sub-elite	11.06 ± 17.06 10.14 ± 16.51	7.31 ± 9.99 9.56 ± 12.60
SE (%)	Elite Sub-elite	88.2 ± 10.18 89.77 ± 7.14	90.21 ± 6.6 89.1 ± 7.05

* Statistically significant difference (*p* < 0.05).

**Table 9:** Athlete supplement use, frequency, average dose and reason for use.

Supplement	Frequency	Dose	Reason	Elite (*n* = 115)	Sub-Elite (*n* = 223)
Caffeine	Daily	100 mg	Performance	23	37
Creatine	Daily	Varied	Performance	13	20
Fish Oil	Daily	1 capsule	Health	18	12
Iron	Daily	Varied	Anaemia/Performance	4	10
Multivitamin	Daily	1 capsule	Health	24	32
Nitrate	Daily	1 shot	Performance	11	1
Probiotics	Daily	1 capsule	Health	13	25
Vitamin D	Daily	1000–4000 IU	Health/Performance	21	5
Whey	Daily	25–40 g	Recovery	22	48
Other (e.g., BCAA, beta—alanine, HMB, casein, antioxidants)	Daily/weekly	Varied	Health/Performance	30	19

**Table 10:** Relationship between supplement use and recovery.

	Sleep Quality	Disturbed Breaks	Emotional Exhaustion	Being in Shape	Self-Efficacy
Supplement Use	−0.167 ** p = 0.002	0.119 * p = 0.029	0.137 * p = 0.012	−0.114 * p = 0.036	−0.108 * p = 0.048

Statistically significant * *p* ≤ 0.05; ** *p* ≤ 0.01.

## Data Availability

The data presented in this study are available on request from the corresponding author.
